# Outbreak of New Delhi metallo-β-lactamase carbapenemase-producing *Pseudomonas aeruginosa* infections in a Southern California hospital

**DOI:** 10.1016/j.ajic.2025.06.005

**Published:** 2025-06-13

**Authors:** Jacob M. Garrigues, Hannah K. Gray, Ricardo P. Ison, Daniel Z. Uslan, Amorce Lima, Shekina Gonzalez-Ferrer, Marisol Trejo, Ran Zhuo, Colette J. Matysiak Match, Erlinda R. Ulloa, Sandeep Bhaurla, Vanessa Lewis, Urvashi Parti, Tessa Sandoval, Sebora Turay, Shaunté Walton, Kavitha Prabaker, Omai B. Garner, Sukantha Chandrasekaran, Nicole M. Green, Shangxin Yang

**Affiliations:** a Public Health Laboratories, County of Los Angeles Department of Public Health, Downey, CA; b Department of Pathology and Laboratory Medicine, David Geffen School of Medicine, University of California, Los Angeles, CA; c Clinical Epidemiology & Infection Prevention, UCLA Health, Los Angeles, CA; d Division of Infectious Diseases, David Geffen School of Medicine, University of California, Los Angeles, CA; e Division of Infectious Diseases, Children’s Hospital of Orange County, Orange, CA; f Department of Pediatrics, University of California Irvine School of Medicine, Irvine, CA; g Acute Communicable Disease Control, County of Los Angeles Department of Public Health, Los Angeles, CA

**Keywords:** NDM, Sink, P-trap, Genomic surveillance, Whole-genome sequencing

## Abstract

**Background:**

Carbapenemase-producing carbapenem-resistant *Pseudomonas aeruginosa* (CP-CRPA) is a major public health threat due to limited treatment options and high transmissibility. Though widespread globally, few instances of locally transmitted New Delhi metallo-β-lactamase (NDM)-producing CP-CRPA have been documented in the United States. Here, a cluster of locally transmitted NDM-1 CP-CRPA infections in Southern California is reported.

**Methods:**

Epidemiologic investigations involving both patient screening and environmental surveillance by microbiological culture, carbapenemase testing, and bacterial whole-genome sequencing were conducted.

**Results:**

Through extensive epidemiologic investigations, a sink environmental reservoir was identified. A genetically unique strain of NDM-CRPA was identified to be transmitted among seven patients in a single hospital over the course of 1.5 years. Changes in antimicrobial resistance genes harbored by a mobile genetic element were identified between the index and later cases, and the gene encoding NDM-1 resided within a mobile genetic element on the chromosome displaying evidence of widespread transmission between different species. Several interventions were implemented, including sink P-trap replacement and disinfectant against *Pseudomonas* biofilms. No additional cases were identified after the interventions.

**Conclusions:**

Implementation of real-time pathogen surveillance enabled effective response to an ongoing outbreak that involved environmental sampling, microbiological testing, whole-genome sequencing, facility management, and updated infection prevention policies.

## BACKGROUND

Carbapenem-resistant *Pseudomonas aeruginosa* (CRPA) accounts for approximately 10% to 30% of clinically isolated *P aeruginosa* strains in the United States.^1^ Carbapenemase production represents only a fraction of this resistance, with the Centers for Disease Control and Prevention reporting 1,530 carbapenemase-producing CRPA (CP-CRPA) among more than 68,000 CRPA isolates collected between 2017 and 2022.^2^ Within CP-CRPA in the United States, Verona Integron-encoded Metallo-β-lactamase (VIM) is the predominant carbapenemase detected. While the New Delhi metallo-β-lactamase (NDM) is prevalent in Asia, Europe, and the Middle East, it remains rare in the United States, with few cases of autochthonous transmission. NDM-producing CP-CRPA can be especially challenging to treat, with ~30% treatment failure.^3^ One such case of NDM-producing CP-CRPA in Southern California is thought to be an endemic strain, given the patient’s lack of international travel and genetic dissimilarity to global strains.^4^

The transmission of carbapenemase genes within and between bacterial populations is of urgent concern, making infection prevention critical to stop the spread of these isolates. A number of outbreaks of CP-CRPA have been reported in health facilities in the United States, including an outbreak of imipenemase metallo-β-lactamase (IMP)-producing strains in an intensive care unit in Idaho,^5^ a cluster of VIM-producing strains in a skilled nursing facility in Chicago,^6^ and a recent report of NDM-1 isolates in Texas, starting with a travel-associated index case.^7^ Certain “high-risk strains” of *P aeruginosa*, such as ST235, ST111, and ST233, are particularly concerning due to their extensive drug resistance (XDR) and carriage of carbapenemase genes. These sequence types, found globally, can harbor both chromosomal and plasmid carbapenemase genes and are associated with over 60 different β-lactamases.^8^

Here, an outbreak involving local transmission of NDM-producing CP-CRPA in a Southern California hospital is described. Epidemiologic investigations and genomic sequencing identified a sink as the environmental reservoir.

## METHODS

### Environmental testing and patient surveillance

A total of 72 environmental samples were collected during the outbreak investigations. Forty-six patients were screened as part of two separate point prevalence surveys. Each patient was screened once, except for one individual with a prolonged admission who was tested during both surveys. The point prevalence surveys were conducted as part of the routine outbreak response protocol to assess potential transmission and identify additional cases.

### Laboratory testing of surveillance and clinical isolates

*P aeruginosa* isolates from clinical specimens exhibiting elevated carbapenem minimum inhibitory concentrations, as determined by microbroth dilution following Clinical and Laboratory Standards Institute (CLSI) guidelines, were screened for carbapenemase production. The NG-test CARBA 5 immunochromatographic lateral flow assay (Hardy Diagnostics) was utilized to detect five carbapenemases: VIM, IMP, NDM, KPC, and OXA-48-like. Between April 2023 and September 2024, eight *P aeruginosa* isolates tested positive for NDM production via this methodology and were included in this study. Clinical isolates were also tested for cefiderocol and imipenem/relebactam susceptibility using Kirby-Bauer disk diffusion according to CLSI guidelines. The environmental surveillance samples (surface swabs) and patient surveillance samples (rectal swabs) were collected using eSwab (Becton Dickinson) and plated on the carbapenem-resistant gram-negative bacteria surveillance culture medium ChromID Carba agar (BioMerieux). Isolates that grew from the surveillance culture were subcultured onto TSA II 5% sheep blood agar (Becton Dickinson) with a meropenem disk to further select for pure carbapenem-resistant isolates, which were then identified by VITEK matrix-assisted laser desorption/ionization time-of-flight mass spectroscopy system (BioMerieux) and tested for carbapenemase production using the Hardy NG-test CARBA 5 assay. Throughout this study, isolate 1 denotes the isolate collected from patient 1, while isolate 2 denotes the isolate collected from patient 2, etc.

### Whole-genome sequencing

Genomic DNA was extracted from bacterial isolates using the Qiagen DNeasy Blood and Tissue extraction kit. For short-read sequencing, libraries were prepared using the Illumina DNA Library Prep kit, followed by paired-end sequencing on a MiSeq instrument using Illumina MiSeq Reagent Kits v2 (500 cycles). For long-read sequencing, libraries were prepared using the Oxford Nanopore Technologies (ONT) Rapid Barcoding Kit and sequenced on an ONT GridION instrument using R10.4 flowcells. Base calling was performed using onboard ONT MinKnow software with the high-accuracy base calling model.

### Genome assembly and genomic analyses

For short-read sequences, de novo assemblies were generated with the TheiaProk_Illumina_PHB v2.2.1 workflow on Terra.bio using default settings.^9^ kSNP3.0 was used to determine core-genome single-nucleotide polymorphism (SNP) differences and construct a maximum-parsimony phylogenetic tree with default settings. Phylogenetic tree visualization was performed using Iroki.^10^ PubMLST^11^ was used to determine multilocus sequence type, and antimicrobial resistance (AMR) genes and point mutations were identified using AMRFinderPlus v3.12.8 with database v2024-05-02.2.^10,12^

Hybrid de novo assemblies combining both short and long reads were generated for isolates obtained from patients 1, 4, and 5 using the TheiaProk_ONT_PHB v2.0.1 workflow on Terra.bio with default settings. Annotations of genes and some noncoding elements (including *attI* sites) for hybrid assemblies were produced using Bakta v1.5.1 with database v4.0,^13^ while AMR genes were identified using AMRFinderPlus. In instances where Bakta and AMRFinderPlus annotations conflicted, AMRFinderPlus annotations superseded. Identification of *attC* sites was performed using HattCI v1.0b with default settings^14^; only *attC* sites identified with a Viterbi score > 1 were considered. Identification of genomic islands containing putative mobile genetic elements (MGEs) was performed using IslandViewer 4.^15^ NCBI BLAST (megablast against database nt) using default parameters^16^ was used to identify homologous regions in reference genomes.

### Research ethics

This study received an IRB exemption; approval was given to perform a chart review of all patients under investigation.

## RESULTS

Eight cases of NDM-producing CP-CRPA were identified in the same Southern California hospital: one from April 2023, five from June through August 2023, one from April 2024, and the last one from September 2024 (Table 1). The index case (patient 1) in this cluster was previously described^4^ and reported recent travel to Hawaii. Patient 2 received recent medical care in Iran, and no other patients reported recent travel outside of the area. Two patients (patients 2 and 6) did not receive any antimicrobial treatment due to asymptomatic bacteriuria, two recovered (patients 1 and 7) after treatment with cefiderocol. Three patients (patients 4, 5, and 8) succumbed due to underlying comorbidities.

Notably, patient 3 was admitted for extracorporeal membrane oxygenation support and initially treated with cefiderocol for NDM-producing CRPA identified in a sputum culture. After stabilizing, the patient was transferred to their home institution for management of acute myeloid leukemia. During this time, the patient developed necrotic skin lesions secondary to calciphylaxis, which became superinfected with multidrug-resistant organisms, including a now pan-resistant NDM-producing CRPA. The patient received bacteriophage therapy, compassionate use of cefepime-zidebactam, and sodium thiosulfate for calciphylaxis. The patient has since fully recovered.

Isolates from these NDM-producing CP-CRPA cases were obtained from various sources, including urine, respiratory tract, blood, and peritoneal fluid (Table 1). Consistent with the production of NDM β-lactamase, phenotypic susceptibility testing (Table 2) found that all isolates were resistant to β-lactam antibiotics, including carbapenems. All isolates included in this study were susceptible to cefiderocol, and those tested for colistin susceptibility exhibited intermediate results.

In response to the identification of NDM-producing CP-CRPA in patients 4 and 5, the first epidemiologic investigation was launched. Commonalities between staff, procedures, equipment, and patient care locations were investigated. From this, it was determined that respiratory therapists (RTs) were shared between four patients, and registered nurses between two patients. All patients were either on a ventilator or had a tracheostomy at the time of their positive culture. However, no RT equipment or machines were identified as shared between patients, and single-use nebulizers and airway clearance devices were strictly used before disposal. Additionally, no shared water sources were identified. Extensive environmental sampling of the RT equipment reprocessing area, including sinks, drains, and work surfaces, did not yield any *P aeruginosa*. As a precaution, the sink in the RT equipment reprocessing area was replaced.

Following identification of the seventh case in April 2024, a point prevalence study was conducted in the unit. While several patients had *P aeruginosa* identified on rectal culture, none were carbapenemase producers. Additional environmental sampling revealed growth of *P aeruginosa* in multiple sinks and P-traps, but none were NDM CP-CRPA. After the eighth case, a third investigation was launched, and extensive environmental sampling ultimately detected two NDM-positive environmental *P aeruginosa* isolates from the drain and P-trap of a sink in an intensive care unit room that previously housed patients 1 and 8.

**Fig. 1.**
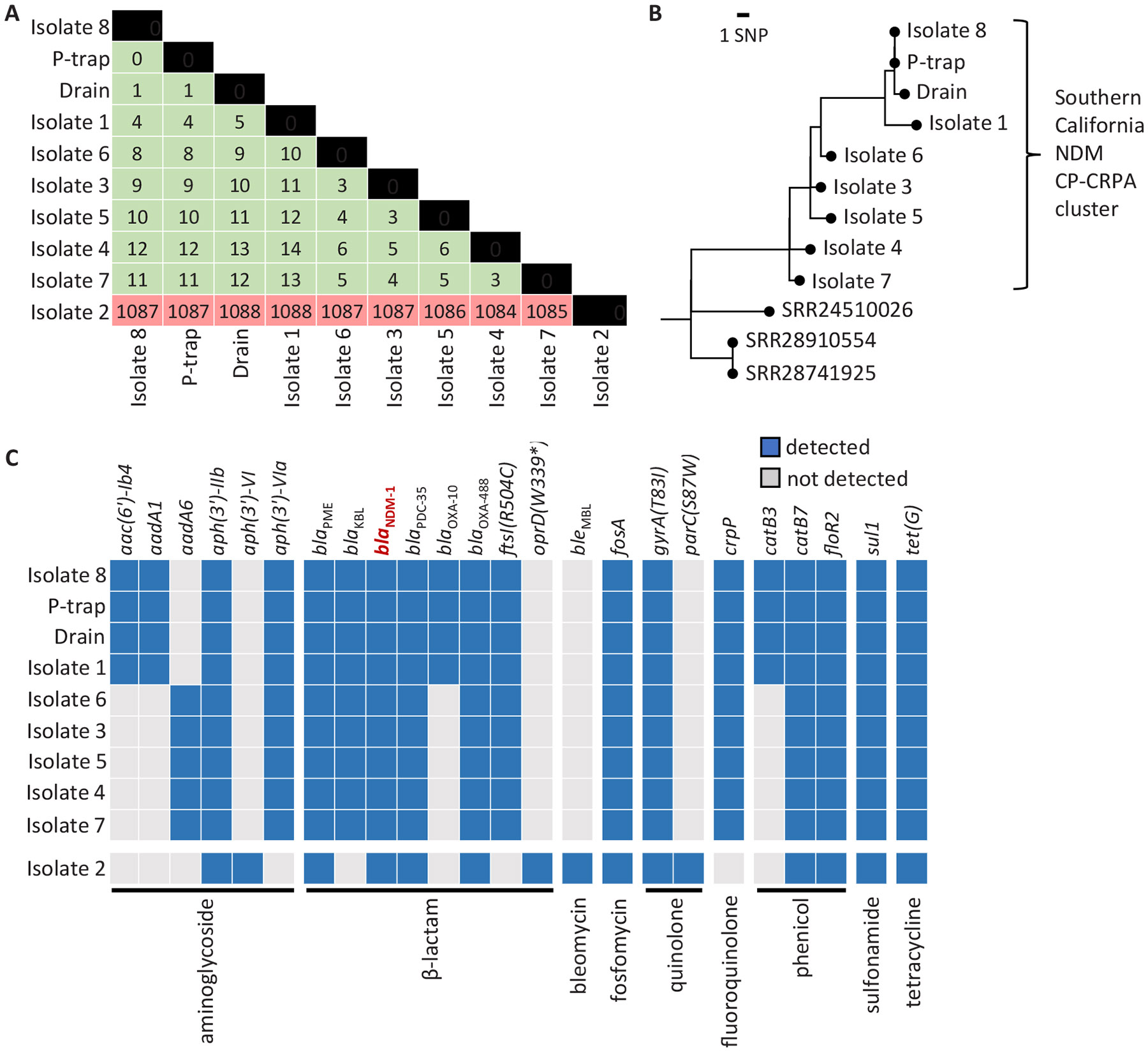
Single-nucleotide polymorphism (SNP) differences, phylogenetic analyses, and antimicrobial resistance (AMR) gene content of Southern California New Delhi metallo-β-lactamase (NDM) carbapenemase-producing carbapenem-resistant *Pseudomonas aeruginosa* (CP-CRPA) isolates. (A) Core-genome SNP differences between Southern California NDM CP-CRPA isolates collected from patients and environmental sources. Isolate 1 was collected from patient 1, while isolate 2 was collected from patient 2, etc. Red denotes a high number of SNP differences, while green represents a low number of SNP differences and isolates likely to be related. SNP differences for isolate 2 were obtained by comparing to all other isolates; pairwise SNP differences for all other isolates were obtained after excluding isolate 2 to maximize core-genome size and the number of detected SNPs. (B) Maximum-parsimony phylogenetic tree based on SNP differences between Southern California NDM CP-CRPA isolates collected from patients and environmental sources as well as similar isolates available at NCBI (SRR28910554, SRR28741925, and SRR24510026). Isolate 2 was excluded due to a high number of SNP differences. Phylogenetic tree was rooted using another similar isolate as an outgroup (SRR13414623, not shown). Scale bar represents SNP distance. (C) AMR genes detected in short-read-based genome assemblies obtained from Southern California NDM CP-CRPA isolates. Isolates were collected from patients and environmental sources. Blue indicates the respective gene was detected, while gray indicates the gene was not detected. The *bla*_NDM-1_ gene is labeled in red. Antimicrobial drug classes are shown.

To determine the relatedness of NDM CP-CRPA isolates from patient and environmental sources, whole-genome sequencing (WGS) using Illumina was performed and the resulting genome assemblies were compared. All isolates were typed as ST235 and harbored the *bla*_NDM-1_ gene. Nucleotide-level comparison of all assemblies revealed that isolate 2 differed by over 1,000 SNPs, indicating it was unrelated to the other isolates (Fig. 1A). After excluding isolate 2, the remaining patient isolates (from patients 1, 3, 4, 5, 6, 7, and 8) and both environmental isolates (sink drain and P-trap) differed by only zero to 14 SNPs, suggesting close relatedness (Fig. 1A). Comparison to other sequenced isolates available at NCBI using Pathogen Detection^17^ revealed that environmental isolates, and those from patients 1, 3, 4, 5, 6, 7, and 8, formed a distinct cluster, separate from other isolates (Fig. 1B). In contrast, isolate 2 did not cluster with any of the other isolates (data not shown). Notably, the isolate collected from patient 8 was most similar to the environmental and the index patient isolates, clustering phylogenetically with both (Fig. 1A,B), consistent with the epidemiologic link of the two patients who stayed in the same room with the contaminated sink, albeit 1.5 years apart. These results suggest a high likelihood that the NDM-producing CP-CRPA was introduced by the index patient and subsequently spread to the other six patients over the course of 1.5 years, and a contaminated sink played an important role in its transmission.

WGS revealed comprehensive AMR gene profiles for individual isolates, consistent with the XDR phenotypes observed. All isolates contained several AMR genes in addition to *bla*_NDM-1_ (Fig. 1C). Notably, isolate 2 harbored aminoglycoside resistance genes *aph(3*′*)-IIb* and *aph(3*′*)-VI*, β-lactamase genes *bla*_PME-1_, *bla*_PDC-35_, and *bla*_OXA-488_, the fosfomycin resistance gene *fosA*, phenicol resistance genes *catB7* and *floR2*, the sulfonamide resistance gene *sul1*, and the tetracycline resistance gene *tet(G)*. Additionally, isolate 2 contained an oprD (W339*) mutation associated with β-lactam resistance, as well as *gyrA(T83I)* and *parC(S87W)* mutations associated with quinolone resistance. Outbreak isolates shared similar AMR gene profiles, including *aph(3*′*)-IIb* and *aph(3*′*)-VIa, bla*_PDC-35_ and *bla*_OXA-488_, *fosA*, the fluoroquinolone resistance gene *crpP*, as well as *catB7*, *floR2*, *sul1*, and *tet(G)*. All outbreak isolates also carried an ambiguous *bla*_PME_ gene, with the closest match being *bla*_PME-1_, a truncated form of the β-lactamase gene *bla*_KBL_, and a penicillin-binding protein 3 gene mutation *ftsI(R504C)* associated with β-lactam resistance. Furthermore, the *gyrA(T83I)* mutation was observed in all outbreak isolates. However, there were some differences observed between outbreak isolates: environmental isolates and those from patients 1 and 8 contained the aminoglycoside resistance genes *aac(6*′*)-Ib4* and *aadA1*, the phenicol resistance gene *catB3*, and the β-lactamase gene *bla*_OXA-10_, while isolates from patients 3, 4, 5, 6, and 7 contained the aminoglycoside resistance gene *aadA6*. These results demonstrate that isolate 2 had a unique AMR gene profile compared with the other isolates, further supporting its unrelatedness. Moreover, the observed AMR gene differences between environmental isolates and those from patients 1 and 8 compared with the remaining isolates suggest that genomic changes emerged over the course of the outbreak.

**Fig. 2.**
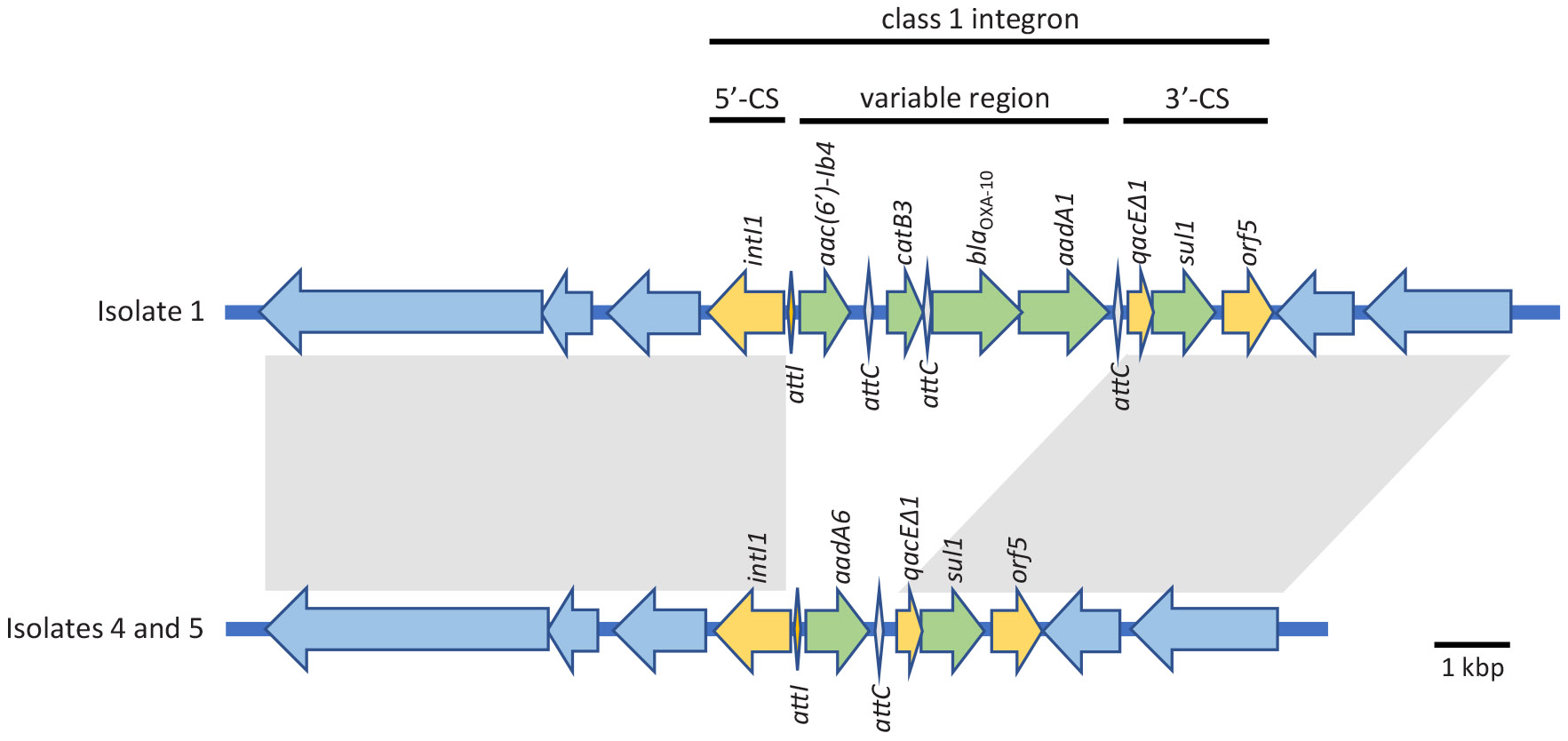
Class 1 integron with antimicrobial resistance (AMR) gene cassette differences between Southern California NDM CP-CRPA isolates. Composition of a class 1 integron containing AMR gene cassettes found in the index and later case isolates. Protein-coding genes are depicted with arrows pointing in the 5′ to 3′ direction. AMR genes are labeled and shown in green, while transposase genes are in blue and other types of genes are in yellow. 5′ and 3′ conserved regions are highlighted (5′-CS and 3′-CS, respectively), which contain the *intl1*, *sul1*, *qacE*Δ*1*, and *orf5* genes. Positions of *attI* and *attC* recombination sites are denoted. Gray shading represents regions shared between isolates. Scale bar represents genomic distance. CP-CRPA, carbapenemase-producing carbapenem-resistant *Pseudomonas aeruginosa*; NDM, New Delhi metallo-β-lactamase.

One potential explanation for the AMR gene differences observed between outbreak isolates may be explained by gene residence within active MGEs. To enable full resolution of MGEs and infer MGE activities occurred during this outbreak, ONT long-read WGS was performed using select isolates and combined with short reads to generate hybrid assemblies. Isolate 1 from the index case contained a class 1 integron nested within a putative Tn*3* family transposon with *sul1* and *qacE*Δ*1* in its 3′ conserved region. Within its variable region, this integron had an array of three gene cassettes (Fig. 2): the first cassette harbored *aac(6*′*)-Ib4*, the second *catB3*, and the third *bla*_OXA-10_ and *aadA1* in different reading frames. In isolates 4 and 5 collected from later cases, a class 1 integron located at the same genomic position contained only one gene cassette that harbored *aadA6*. Taken together, these observations suggest that the variability in AMR genes and phenotypic susceptibility observed within this outbreak was due to gene cassette switching in an integron that remained in place.

**Fig. 3.**
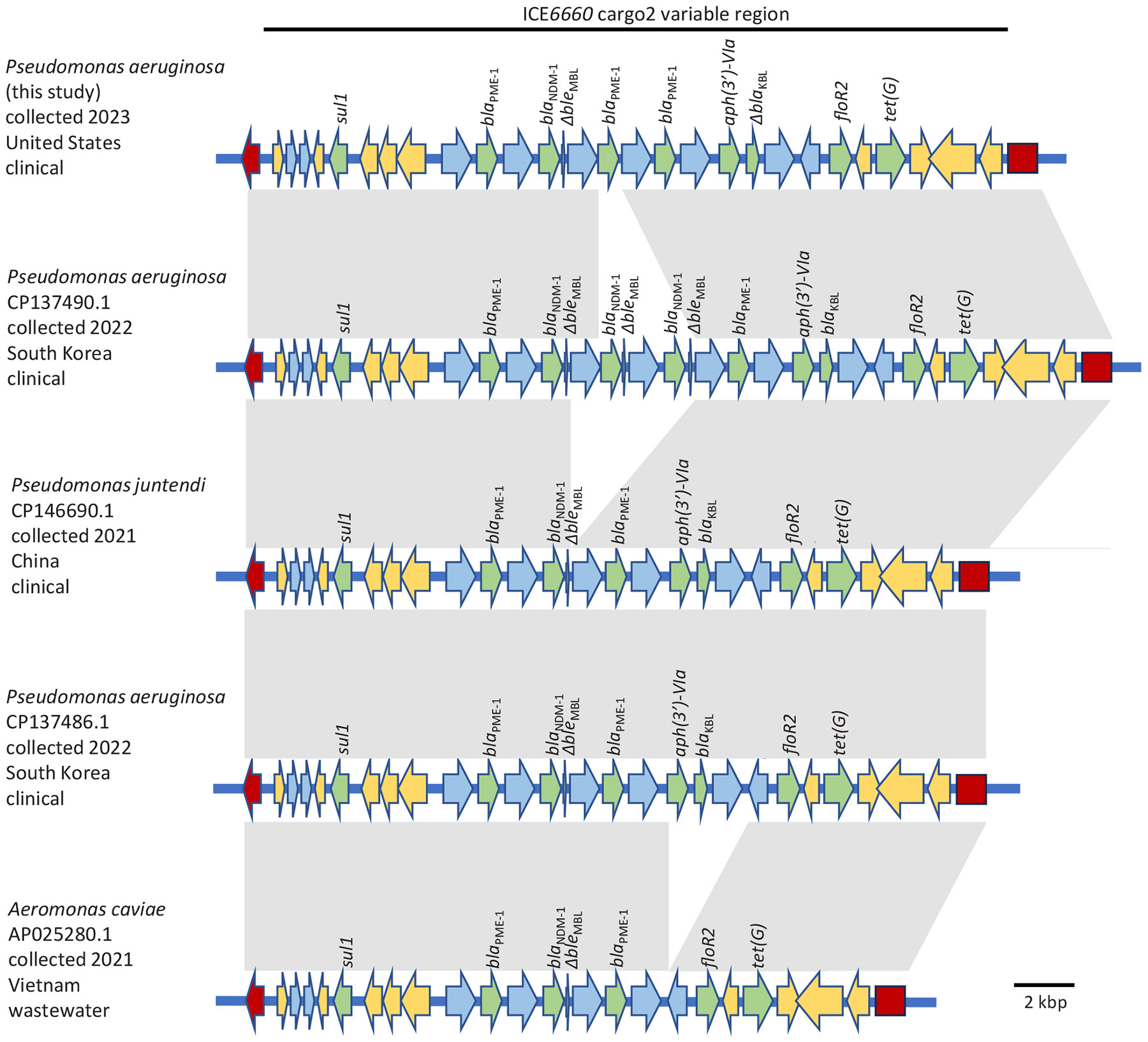
Antimicrobial resistance (AMR) gene arrays carried by ICE*6660*-like elements found in Southern California New Delhi metallo-β-lactamase (NDM) carbapenemase-producing carbapenem-resistant *Pseudomonas aeruginosa* (CP-CRPA) isolates and other unrelated isolates. Genes detected in cargo2 variable regions nested within ICE*6660*-like elements identified in hybrid genome assemblies obtained from Southern California NDM CP-CRPA isolates and other publicly available assemblies. Protein-coding genes are represented with arrows pointing in the 5′ to 3′ direction. AMR genes are labeled and shown in green, while transposase genes are in blue and other types of genes are in yellow. Depicted in red is a DNA methyltransferase gene where the cargo2 variable region has been inserted. Gray shading represents regions shared between isolates. Scale bar represents genomic distance.

We also explored the intraspecific transmissibility of the *bla*_NDM-1_-containing MGE. Hybrid genome assemblies of Southern California CP-CRPA isolates revealed that *bla*_NDM-1_ in outbreak isolates was located within a chromosomal array of AMR genes containing *sul1*, three copies of *bla*_PME-1_, *aph(3*′*)-VIa*, *floR2*, and *tet(G)*, as well as truncated forms of *bla*_KBL_ and *ble*_MBL_, which was not detected in short-read-based assemblies. This array was located within the "cargo2" region of a previously described MGE, integrative and conjugative element (ICE) *6660*^18^ (Fig. 3). This ICE*6660* cargo2 region resembled others found in sequenced isolates available at NCBI, including *P aeruginosa* from South Korea, *P juntendi* from China, and *Aeromonas caviae* from Vietnam (Fig. 3), suggesting widespread exchange across species and even genera.

## DISCUSSION

Sequencing revealed close genetic similarity (0-14 SNPs) between all isolates, except isolate 2, which was quickly determined to be unrelated. Given the high prevalence of NDM-1 CP-CRPA in Iran and the Middle East,^19,20^ this isolate may be associated with the patient’s extensive health care history in the region prior to visiting the Southern California facility. A prior study demonstrated that the index isolate, isolate 1, was distinct from previously described global isolates, indicating it was likely a unique domestic strain.^4^ The subsequent isolates are also likely descendants of this same strain, given a *P aeruginosa* mutation rate of between 6 × 10^−4^ and 1.6 × 10^−1^ mutations per genome per generation.^21^ Indeed, phylogenetic analyses using the National Center for Biotechnology Information (NCBI) Pathogen Detection Isolates Browser^17^ demonstrated that all the Southern California isolates distinctly cluster together relative to other isolates available at NCBI, indicating a hospital outbreak that was originated from an index patient without international travel history, and transmitted nosocomially including via a contaminated sink. The outbreak progressed slowly, and cases were identified sporadically, spanning 1.5 years. The insidious nature of the transmission, despite exhaustive investigation and numerous negative environmental cultures, made the investigations challenging. The source was not identified until the third intensive culture–based investigation with dozens of environmental samples tested.

MGEs such as plasmids, transposons, and ICEs are major contributors to the spread of AMR genes through horizontal gene transfer.^22^ Notably, the NDM-1 gene is located on the chromosome and not on a plasmid, which makes this strain inherently resistant to carbapenems. No plasmids were detected in isolates from this outbreak, while multiple AMR genes were found within putative MGEs inserted into the bacterial chromosome. Several AMR genes, including *bla*_NDM-1_, were found in an array within the cargo2 region of an ICE*6660* element; 3 copies of *bla*_PME-1_ were present. This region is similar to other ICE*6660* cargo2 regions found in clinical *P aeruginosa* and *P juntendi* isolates collected in South Korea and China, respectively, as well as an environmental *A caviae* isolate collected from wastewater in Vietnam, suggesting widespread horizontal gene transfer. Chromosomal insertions suggest AMR genes in the Southern California CP-CRPA strain may be more stable than if they were located on plasmids, which heightens the epidemiologic significance of this XDR pathogen.

The observed differences in detected AMR genes between genetically related isolates suggested some AMR genes were lost and acquired within this cluster. Integrons are genetic elements containing a variable region flanked by *attI* and *attC* recombination sites allowing for the capture and exchange of different gene cassettes, and can be carried by transposons integrated into the genome or plasmids.^23^ The isolate from the index case within this outbreak had a class 1 integron containing three AMR gene cassettes harboring *aac (6*′*)-Ib4*, *catB3*, *bla*_OXA-10_, and *aadA1*, while isolates from later cases had only one AMR gene cassette with *aadA6*. As these integrons were inserted at the same location on the bacterial chromosome, these findings suggest that AMR gene cassettes were swapped during the course of this outbreak, providing an example of AMR gene dynamics that can occur in related isolates. Therefore, phenotypic susceptibility results should be interpreted with caution for outbreak investigations.

Nosocomial spread of *Pseudomonas* via sinks has been demonstrated in numerous studies, as the bacteria are well-adapted to thrive in moist environments and can form a biofilm extending from the P-trap to the drain.^24-27^ Based on our investigations and findings, several interventions were implemented, including: (1) replacing the sink P-trap in the affected room; (2) weekly application of an EPA-registered disinfectant against *Pseudomonas* biofilms (Virasept, Ecolab, Inc) in all sinks; (3) staff education; (4) installation of splash guards in the medication preparation areas; (5) reminders to staff to keep patient care supplies out of sink splash zones; (6) adjusting faucets so they do not pour directly on the drain; (7) regular audits of environmental services cleaning; and (8) use of the CDC Water Infection Control Risk Assessment (WICRA) for Healthcare Settings to evaluate and prioritize water risk interventions. Follow-up environmental cultures obtained six weeks later, did not detect NDM CP-CRPA. No additional cases were identified as of the submission of this paper (February 24, 2025).

While the contaminated sink was strongly implicated in the transmission of NDM-producing CP-CRPA to patients 1 and 8, not all cases in this outbreak could be directly epi-linked to that environmental reservoir. For instance, patients 4 and 5 were identified on the same date (July 7, 2023). Although they were not housed in the same room or hospitalized concurrently, our review of care patterns revealed overlapping involvement of respiratory therapy and nursing staff, as well as shared use of procedural rooms for similar interventions. These observations raise the possibility of indirect cross-transmission via health care personnel or contaminated procedural environments. While causality cannot be confirmed retrospectively, such overlaps represent plausible transmission routes that merit consideration in outbreak investigations. This highlights the complexity of health care-associated pathogen transmission and underscores the importance of evaluating both environmental and operational factors in prolonged outbreak responses. Contaminated sink drains have been clearly linked to numerous outbreaks of infections due to gram-negative bacilli, including *Pseudomonas*, and have received increasing attention as an environmental reservoir.^28^ Colonized drains can result in intermittent dispersal of organisms to other environmental surfaces such as countertops, to health care worker or patient hands or clothing, or medical equipment.^29-31^ In outbreaks similar to this one, prolonged durations have been noted lasting many months or years. Longer patient length of stay may be a risk factor for patient acquisition.^25,32^ Once established, eradication of sink drain colonization is challenging due to persistence of organisms in biofilms and inadequate contact time achieved by disinfectants when poured into a sink drain. In this outbreak, the lengthy time in-between cases and initially negative environmental cultures highlight the challenges with outbreak investigation of potentially contaminated sinks. Intensive water management interventions, such as application of EPA-registered disinfectant with biofilm claims, may be required to control transmission.^25,31,32^ Sink drain and wastewater plumbing should be included in health care water infection control risk assessments and investigated as a potential source of outbreaks due to gram-negative bacilli.^25,32^

## CONCLUSIONS

NDM-producing CRPA poses an extremely high risk to patients, especially those with reduced immune function and extended hospitalization. Use of WGS contributed to the rapid detection, characterization, and subsequent infection control response, which is critical to curbing the spread of these organisms in health care settings. We demonstrate that a unique strain of NDM CP-CRPA was transmitted among seven patients in a single hospital over the course of 1.5 years, with two cases linked to a contaminated sink. Implementation of real-time pathogen surveillance enabled effective response to an ongoing outbreak that involved environmental sampling, microbiological testing, WGS, facility management, and updated infection prevention policies.

## Figures and Tables

**Table 1:** Clinical history of Southern California NDM-producing *Pseudomonas aeruginosa* cases

Patient	Isolate recovery date	Age	Travel history	Significant comorbidities and medical history	Isolate source	Treatment	Outcome
1	04/07/2023	50	Domestic travel (Hawaii)	End-stage cardiac disease, cardiogenic shock, veno-arterial extracorporeal membrane oxygenation (ECMO), and orthotopic heart transplant (OHT)	Urine	Cefiderocol	Discharged
2	06/04/2023	84	Iran, extended medical care	Parkinson’s disease, hip fracture, and replacement	Urine	Untreated	Discharged
3	07/04/2023	19	None noted	Acute myeloid leukemia, acute hypoxic respiratory failure requiring ECMO	Respiratory, BAL	Cefiderocol, ceftazidime-avibactam, aztreonam, azithromycin, tobramycin, cefepime-zidebactam, and phage therapy	Discharged
4	07/07/2023	74	None noted	Prostate cancer, sepsis, enterocolitis, *Clostridium difficile* infection, severe acute kidney injury, aspiration pneumonia, acute hypoxemic respiratory failure, pulmonary edema, and multiorgan dysfunction syndrome	Blood	Untreated	Deceased
5	07/07/2023	76	None noted	Papillary thyroid cancer, radiation therapy, tracheal hemorrhage, and gangrenous cholecystitis	Respiratory, tracheal aspirate	Cefiderocol	Deceased
6	08/01/2023	67	None noted	Nonischemic cardiomyopathy, atrial fibrillation, cardiogenic shock, and OHT	Urine	Untreated	Discharged
7	04/24/2024	54	None noted	Heart failure, ECMO, OHT, and seroma at the ECMO cannulation	Body fluid, abdomen	Cefiderocol	Discharged
8	09/23/2024	38	None noted	Metastatic thymoma, sterno-thoracotomy with radical thymectomy, en bloc extrapleural pneumonectomy, pericardial reconstruction, pneumothorax with chest tube placement, ECMO, and ventilator-associated pneumonia	Respiratory, tracheal aspirate	Cefiderocol	Deceased

*NDM*, New Delhi metallo-β-lactamase.

**Table 2:** Antibiotic susceptibility phenotypes of Southern California NDM-positive *Pseudomonas aeruginosa* isolates from patients

Drug class	Drug	Isolate 1	Isolate 2	Isolate 3	Isolate 4	Isolate 5	Isolate 6	Isolate 7	Isolate 8
Aminoglycoside	Amikacin	Intermediate	Resistant	Susceptible	Intermediate	Susceptible	Resistant	Intermediate	Intermediate
	Gentamicin	Resistant	Resistant	Resistant	Resistant	Resistant	Resistant	Resistant	Resistant
	Tobramycin	Resistant	Resistant	Susceptible	Susceptible	Susceptible	Susceptible	Susceptible	Resistant
β-lactam								
Cephalosporin	Cefepime	Resistant	Resistant	Resistant	Resistant	Resistant	Resistant	Resistant	Resistant
	Ceftazidime	Resistant	Resistant	Resistant	Resistant	Resistant	Resistant	Resistant	Resistant
β-lactam/β-lactamase inhibitor	Amox/Clav	Resistant	Resistant	Resistant	Resistant	Resistant	Resistant	Resistant	Resistant
	Ceftaz/Avi	Resistant	Resistant	Resistant	Resistant	Resistant	Resistant	Resistant	Resistant
	Cefto/Tazo	Resistant	Resistant	Resistant	Resistant	Resistant	Resistant	Resistant	Resistant
	Imi/Rele	Resistant	Resistant	Resistant	Resistant	Resistant	Resistant	Resistant	Resistant
	Pip/Tazo	Resistant	Resistant	Resistant	Resistant	Resistant	Resistant	Resistant	Resistant
Carbapenem	Imipenem	Resistant	Resistant	Resistant	Resistant	Resistant	Resistant	Resistant	Resistant
	Meropenem	Resistant	Resistant	Resistant	Resistant	Resistant	Resistant	Resistant	Resistant
Siderophore cephalosporin	Cefiderocol	Susceptible	Susceptible	Susceptible	Susceptible	Susceptible	Susceptible	Susceptible	Susceptible
Fluoroquinolone	Ciprofloxacin	Resistant	Resistant	Resistant	Resistant	Resistant	Resistant	Resistant	Resistant
Polymyxin	Colistin	Intermediate	Intermediate	Intermediate	Intermediate	Not tested	Not tested	Intermediate	Intermediate
Sulfonamide	Trimeth/Sulf	Resistant	Resistant	Resistant	Resistant	Resistant	Resistant	Resistant	Resistant

*Amox/Clav*, Amoxicillin/Clavulanic acid; *Ceftaz/Avi*, Ceftazidime/Avibactam; *Cefto/Tazo*, Ceftolozane/Tazobactam; *Imi/Rele*, Imipenem/Relebactam; *NDM*, New Delhi metallo-β-lactamase; *Pip/Tazo*, Piperacillin/Tazobactam; *Trimeth/Sulf*, Trimethoprim/Sulfonamide.
